# Virulence-associated genes and antibiotic resistance patterns of *Vibrio* spp. isolated from cultured marine fishes in Malaysia

**DOI:** 10.1186/s12917-019-1907-8

**Published:** 2019-05-28

**Authors:** Nurliyana Mohamad, Mohammad Noor Azmai Amal, Mohd Zamri Saad, Ina Salwany Md Yasin, Nor Amalina Zulkiply, Muskhazli Mustafa, Nurrul Shaqinah Nasruddin

**Affiliations:** 1 Department of Biology, Faculty of Science, Universiti Putra Malaysia, 43400 UPM Serdang, Selangor Malaysia; 2 Department of Veterinary Laboratory Diagnosis, Faculty of Veterinary Medicine, Universiti Putra Malaysia, 43400 UPM Serdang, Selangor Malaysia; 3 Department of Aquaculture, Faculty of Agriculture, Universiti Putra Malaysia, 43400 UPM Serdang, Selangor Malaysia; 4 Laboratory of Marine Biotechnology, Institute of Bioscience, Universiti Putra Malaysia, 43400 UPM Serdang, Selangor Malaysia; 5 Centre for Craniofacial Diagnostics and Biosciences, Faculty of Dentistry, Universiti Kebangsaan Malaysia, Jalan Raja Muda Abdul Aziz, 50300 Kuala Lumpur, Malaysia

**Keywords:** *Vibrio*, Cultured fish, Virulence genes, Multiple antibiotics resistance

## Abstract

**Background:**

Vibriosis is an important bacterial disease of cultured marine fishes worldwide. However, information on the virulence and antibiotic resistance of *Vibrio* spp. isolated from fish are scarce. This study investigates the distribution of virulence associated genes and antibiotic resistance patterns of *Vibrio* spp. isolated from cage-cultured marine fishes in Malaysia.

**Results:**

A total of 63 *Vibrio* spp. isolated from 62 cultured marine fishes in various geographical regions in Peninsular Malaysia were analysed. Forty-two of the isolates (66.7%) were positive for all *chiA*, *luxR* and *vhpA*, the virulence genes produced by pathogenic *V. harveyi*. A total of 62 *Vibrio* isolates (98%) had *tlh* gene of *V. parahaemolyticus*, while *flaC* gene of *V. anguillarum* was detected in 43 of isolates (68%). Other virulence genes, including *tdh*, *trh*, *hlyA* and *toxR*_vc_ were absent from any of the isolates. Multiple antibiotic resistance (MAR) was exhibited in all strains of *Harveyi* clade, particularly against ampicillin, penicillin, polypeptides, cephems and streptomycin. The MAR index ranged between 0.06 and 0.56, and 75% of the isolates have MAR index of higher than 0.20. Host species and geographical origin showed no correlation with the presence of virulence genes and the antibiotic resistance patterns of *Vibrio* spp.

**Conclusions:**

The study indicates that majority of *Vibrio* spp. isolated from cultured marine fishes possess virulence genes, but were not associated with human pathogen. However, the antibiotics resistance is a real concern and warrants ongoing surveillance. These findings represent an updated knowledge on the risk of *Vibrio* spp. to human health, and also provides valuable insight on alternative approaches to combat vibriosis in cultured fish.

## Background

*Vibrio* spp. that have been associated with diseases in animals and human often possess virulence factors, which are not available or present in the environmental *Vibrio* [1]. However, since *Vibrio* possesses highly plastic genome, the probability of horizontal transfer of the virulence genes between pathogenic and environmental *Vibrio* is high. This contributes to the increased number of pathogenic *Vibrio* strains in aquatic environment [2]. Recently, more disease outbreaks following infections by *Vibrio harveyi*, *V. alginolyticus, V. parahaemolyticus* and *V. campbellii* in farmed fishes were reported in many tropical countries [3–6].

Several extracellular products that are known to contribute to the virulence of *Vibrio* include proteases, hemolysins, phospholipases, siderosphores, cytotoxins, biofilm formation, quorum sensing, and presence of phage [7–9]. Swarming motility of *Vibrio* has been consistently associated with their virulence [10], while hemolysin is a common virulence factor reported in *Vibrio* associated with both fish and human diseases [11]. In addition, virulence of several pathogenic *Vibrio* has also been attributed to quorum-sensing, the bacterial cell to cell communication [12].

Resistance to the bactericidal mechanisms is another important contributor to the virulence of fish pathogen. In the past few decades, antimicrobial resistance has emerged and evolved in *Vibrio* spp. due to the excessive use of antibiotics in human medicine, agriculture and aquaculture systems [13]. This issue gained great concern due to the increased resistance of pathogenic *V. parahemolyticus*, *V. harveyi* and *V. vulnificus* towards many clinically used antimicrobials [14–18]. Moreover, multiple antibiotic resistance (MAR) strains of *V. harveyi* and *V. alginolyticus* have caused severe economic setbacks to the aquaculture industry [19].

This study described the presence of virulence-associated genes and antibiotic resistance patterns of *Vibrio* spp. within the *Harveyi* clade, which were isolated from various aquaculture areas in Peninsular Malaysia. Three typical virulence genes that were possessed by *V. harveyi* (*chiA*, *luxR* and *vhpA*) and five atypical virulence genes that contributed to pathogenic *Vibrio* of both fish and human (*flaC*, *hlyA*, *toxR*_*vc*_, *tdh* and *trh*) were targeted. Furthermore, thermolabile hemolysin gene *tlh*, a species specific marker for *V. parahaemolyticus* was also included. In addition, resistance of the isolates towards 16 commercial antibiotics of various groups were determined to evaluate the potential responsiveness to the suite of antibiotic treatments that most frequently used in aquaculture.

## Results

**Fig. 1:**
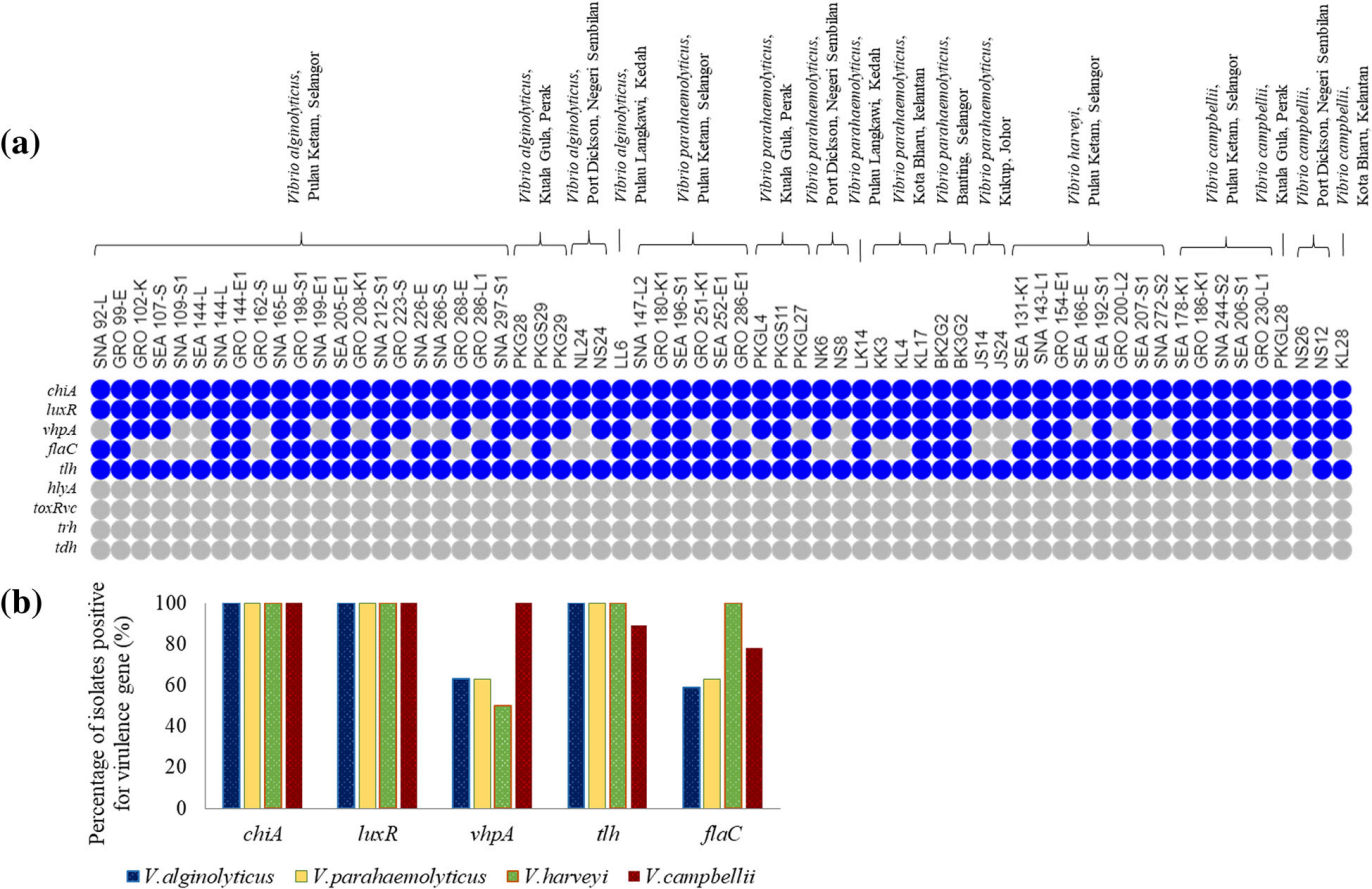
The distribution pattern of virulence genes in isolates of *Harveyi* clade in this study. (**a**) The presence (blue dot) and the absence (grey dot) of target genes in *Vibrio* isolates according to their species; (**b**) The summary of *Vibrio* spp. positive with *chiA*, *luxR*, *vhpA*, *tlh* and *flaC*

Generally, five out of the nine targeted virulence genes were present in the tested isolates (Fig. 1a). All (100%) 63 isolates of *Harveyi* clade possessed typical virulence genes of *chiA* and *luxR*. Forty-two isolates (67%) of studied *Harveyi* clade and all (100%) *V. campbellii* isolates were positive of *vhpA* gene. However, only two out of six virulence genes were detected in other *Vibrio* spp. tested in this study. The *tlh* was detected in all isolates except an isolate of *V. campbellii*.

The *flaC* gene was detected in 68% of the isolates (Fig. 1b). All *V. harveyi* and *V. parahaemolyticus* that were isolated from Pulau Ketam were positive for *flaC*. However, they lack the *tdh*, *trh*, *hlyA* and *toxR*_vc_ genes. Moreover, 27 (43%) of the isolates carried all the virulence genes of *chiA*, *luxR*, *vhpA*, *flaC* and *tlh*, including all *V. campbellii* that were isolated from Pulau Ketam, and two *V. parahaemolyticus* from Banting. However, there was no correlation between the presence of virulence genes and the source of the isolates.

**Fig. 2:**
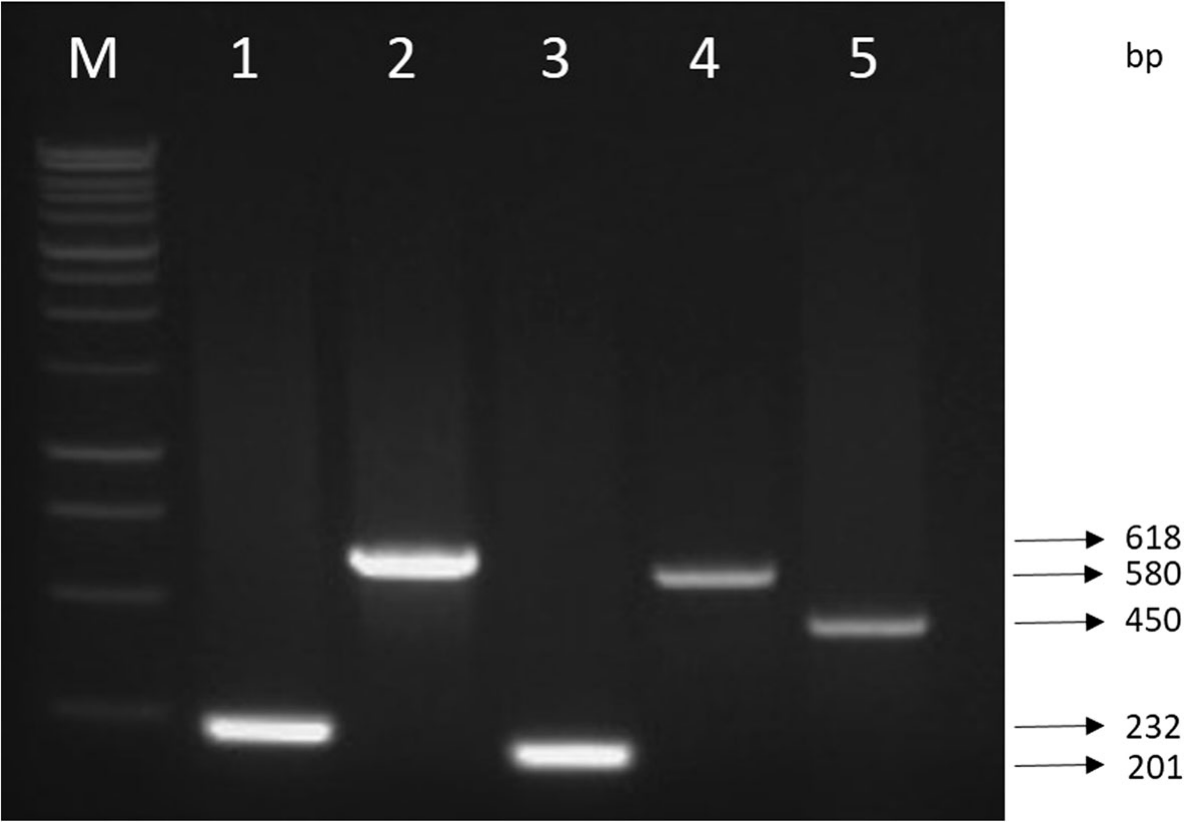
Presence of the *chiA*, *luxR*, *vhpA* and *flaC* of *V. anguillarum* and *tlh* of *V. parahaemolyticus* in isolates of *Harveyi* clade. Lane M: 1 kb DNA ladder; Lane 1: *chiA* (232 bp); Lane 2: *luxR* (618 bp); Lane 3: *vhpA* (201 bp); Lane 4: *flaC* (580 bp) and Lane 5: *tlh* (450 bp)

**Fig. 3:**
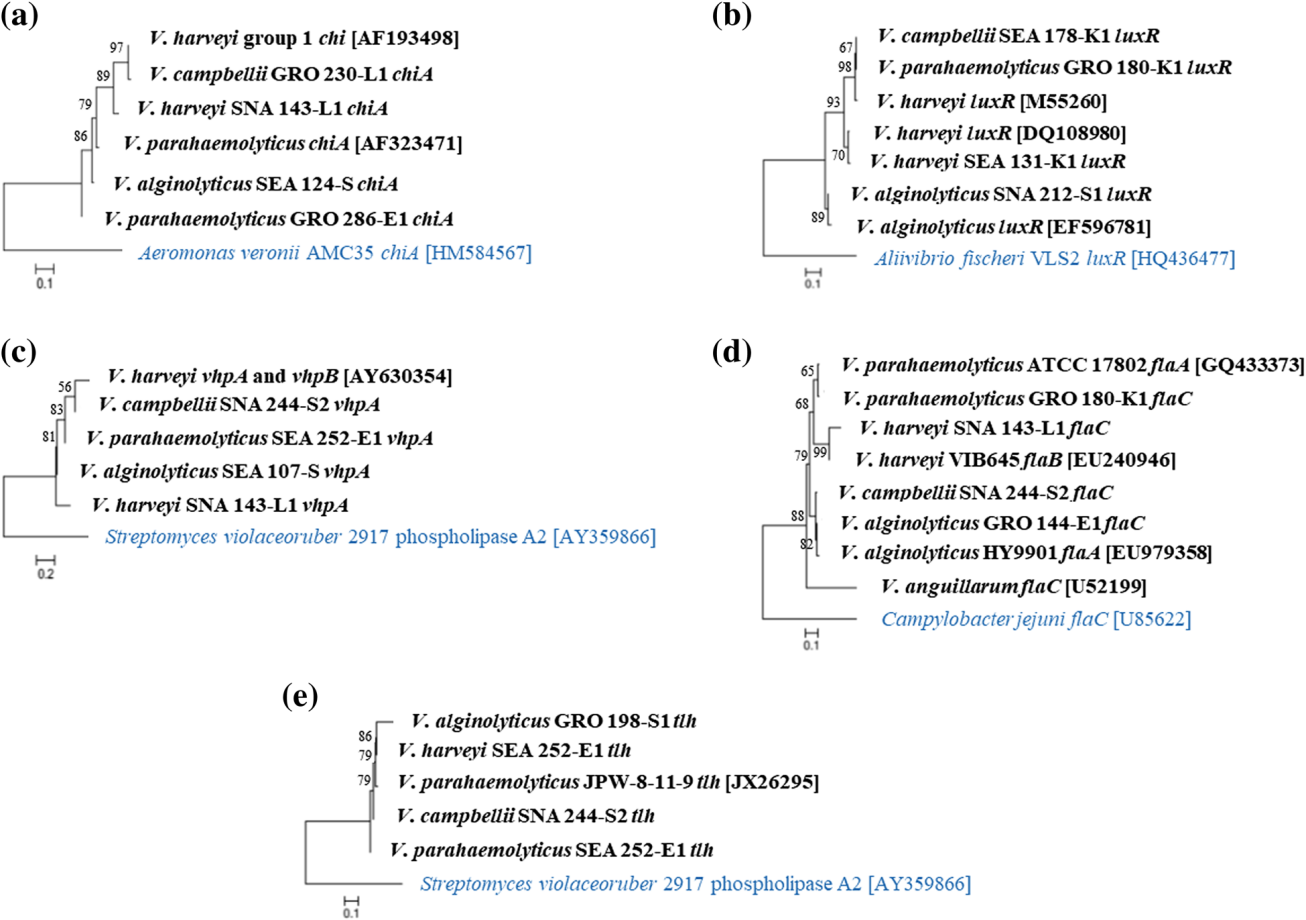
Neighbour-joining tree (Kimura 2-parameter model) of (**a**) *chiA* (**b**) *luxR* (**c**) *vhpA* (**d**) *flaC* (**e**) *tlh* gene sequences from representative *Vibrio* isolates in this study. Reference sequences acquired from the NCBI GenBank are marked with their accession numbers. Strains in blue colour served as outgroup

Amplification of *chiA*, *luxR*, *vhpA*, *flaC* and *tlh* from all species of *Vibrio* isolates, yielded products of approximately 232 bp, 618 bp, 201 bp, 580 bp, and 450 bp, respectively (Fig. 2). Figure 3 shows the phylogenetic tree of the virulence genes. Sequence analysis revealed that the *chiA* of *V. campbellii* GRO 230-L1 and *V. harveyi* SNA 143-L1 shared 99 and 89% similarity, respectively with *chiA* of *V. harveyi*, while *V. alginolyticus* SEA 124-S and *V. parahaemolyticus* GRO 286-E1 showed 95–99% similarity with chitinase A from *V. parahaemolyticus* [AF323471]. The *luxR* of *V. harveyi* SEA 131-K1, *V. campbellii* SEA 178-K1 and *V. parahaemolyticus* GRO 180-K1 were > 98% similar with *luxR* of *V. harveyi*. However, *luxR* of *V. alginolyticus* SNA 212-S1 was identical (99%) to *luxR* of *V. alginolyticus* [EF596781]. All *vhpA* in this study shared high similarity (> 98%) with *vhpA* of *V. harveyi*. Similarly, high similarity (> 89%) was also observed between *tlh* of *Vibrio* isolates in this study and *tlh* of *V. parahaemolyticus* JPW-8-11-1. In addition, *flaC* of *V. harveyi* SNA 143-L1 and *V. alginolyticus* GRO 144-E1 were highly identical (99%) with *flaA* of *V. alginolyticus* HY9901. On the other hand, *flaC* of *V. parahaemolyticus* shared 99% similarity with *flaA* of *V. parahaemolyticus* ATCC 17802, while *flaC* of *V. harveyi* SNA 143-L1 shared 96% with *flaB* of *V. harveyi* VIB645.

**Fig. 4:**
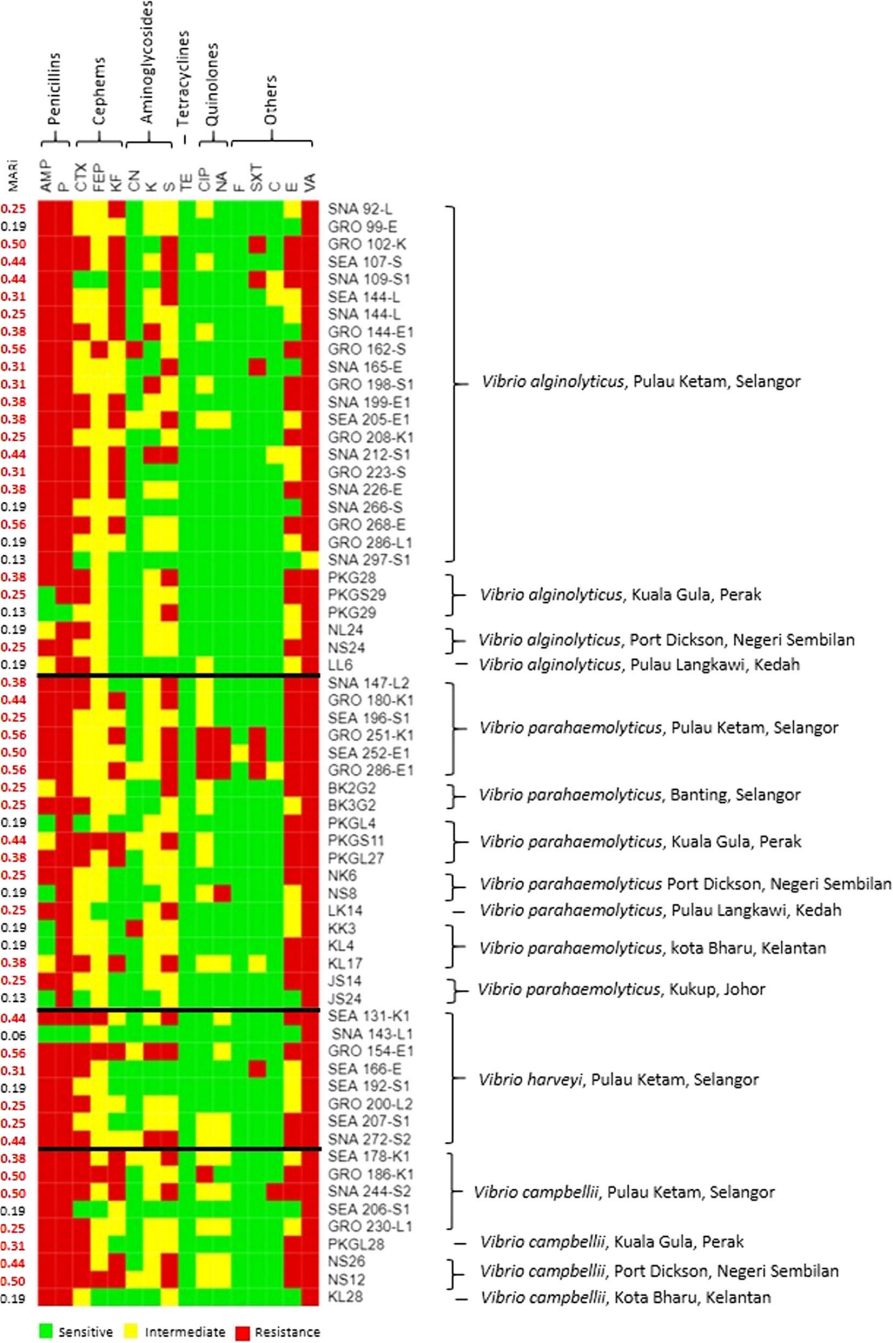
Antibiotic resistance patterns showed by isolates of *Harveyi* clades isolated from sampled fishes. MAR index (MARi) indicated the multiple antibiotics resistance index for the isolates. AMP: Ampicillin (10 μg), P: Penicillin G (10 unit), CTX: Cefotaxime (30 μg), FEP: Cefepime (30 μg), KF: Cephalothin (30 μg), CN: Gentamycin (10 μg), K: Kanamycin (30 μg), S: Streptomycin (10 μg), TE: Tetracycline (30 μg), CIP: Ciprofloxacin (5 μg), NA: Nalidixic acid (30 μg), F: Nitrofurantoin (300 μg), SXT: Sulfamethoxazole/trimethoprim (1.25/23.75 μg), C: Chloramphenicol (30 μg), E: Erythromycin (15 μg), VA: Vancomycin (30 μg)

The antibiotic resistance patterns are illustrated in Fig. 4. Highest resistance (79 to 99%) was observed against penicillin and the polypeptides group of antibiotics. All *Vibrio* spp. isolated from Pulau Ketam, with the exception of one isolate of *V. alginolyticus* and *V. harveyi* were resistance against AMP, P and VA. This was also exhibited by all *V. campbellii* isolates, regardless of their geographical origin. On the other hand, sensitivity towards AMP was mostly observed in *V. parahaemolyticus* isolated from Perak, Kedah, Kelantan and Johor. High resistance towards E was also observed in this study, where only 9% of the isolates showed sensitivity towards E.

A total of 60 and 46% of isolates were resistance against at least one antibiotic from cephems and aminoglycosides groups. Lower resistance level was observed for CN, K and FEP, with less than 6% resistant isolates. However, more than 40% of the tested isolates were resistant against CTX, KF and S. The resistance against CTX was mostly showed by *V. campbellii* (78%), followed by *V. harveyi* (63%) and *V. alginolyticus* (62%). Low resistance (8%) towards quinolones group of antibiotic was observed in this study where none of *V. alginolyticus* and *V. harveyi* isolate was resistant towards CIP and NA. In addition, only one and seven isolates were resistant to C and SXT, respectively. None of the isolate in this study was resistant against F and TE.

The MAR index denotes the extent of environmental contamination by antimicrobial agents which potentially harmful to human health [20]. A MAR index higher than 0.2 indicated high-risk exposure to antibiotics. In this study, the MAR index ranged between 0.06 and 0.56. Approximately 75% of the isolates showed MAR index of higher than 0.20, including 80% from Pulau Ketam. However, no clear pattern was observed between the MAR index and the geographical origin. Nevertheless, an isolate of *V. harveyi* and two isolates of *V. parahaemolyticus* showed resistance against the nine antibiotics tested, with MAR index of 0.56. Among the three isolates, *V. parahaemolyticus* (GRO 286-E1) isolated from Pulau Ketam exhibited strongest resistance towards antibiotics tested, where only two antibiotics (TE and F) can inhibit the growth of this isolate.

## Discussion

This study examines two factors that contribute to the pathogenicity of *Vibrio* spp., which previously isolated from cultured marine fishes in selected important farms and hatcheries in Peninsular Malaysia; the presence of virulence genes associated with pathogenic strains and the antibiotics resistance of *Vibrio* spp.

Virulence genes including *chiA*, *luxR*, *toxR*_*vh*_, *vhpA*, serine protease and *vhh* were widely distributed among pathogenic *V. harveyi* [21]. The distribution of these virulence genes in the closely related species of *V. harveyi* has also been documented [7]. In agreement to previous studies, *chiA* and *luxR* were found to presence in all isolates of *Harveyi* clade analysed in present study.

On the other hand, typical virulence gene of *vhpA* was observed in > 50% of the isolates in this study, with 100% prevalence in *V. campbellii* isolates. Even though *vhpA* was reported as a typical virulence gene harboured by *V. harveyi*, only 50% of our *V. harveyi* isolates was positive for this gene. Ruwandeepika et al. [21], reported a 100% prevalence of *vhpA* among *V. harveyi* but conversely, *vhpA* was reported to be absent in all *V. harveyi* isolated from diseased cultured fish in China, regardless of the virulence level [2, 19].

Studies demonstrated that the presence of chitinase and metalloprotease in ECP of *Vibrio* can cause disease in wide range of aquatic animals including fish, oyster and shrimp [7, 22]. In addition, quorum sensing was reported to regulate the production of these ECP and other virulence genes in Gram-negative fish pathogens [12]. For instance, Defoirdt [23] described on the virulence of *V. harveyi* controlled by quorum sensing. Another study by Croxatto et al. [24] demonstrated the involvement of quorum sensing in the secretion of metalloprotease *EmpA* and biofilm formation in *V. anguillarum*.

In this study, *flaC* was widely distributed in 60% of *V. alginolyticus*, 63% of *V. parahaemolyticus*, and 78% *V. campbellii* isolates. In addition, all *V. harveyi* harboured *flaC* gene. In a study conducted by Bai et al. [25], 37.5% of the *V. harveyi* isolates carried the *flaC* gene. They also reported that the *flaC* gene was widely distributed in other *Vibrio* spp. including *V. anguillarum*, *V. alginolyticus*, *V. campbellii*, *V. fischeri*, *V. fluvialis*, *V. mimicus*, *V. natriegens* and *V. parahaemolyticus*. Another study revealed that *flaC* was detected in 60% of the *Vibrio* in the *Harveyi* clade [21]. Similarly, the sequence diversity of *flaC* observed in this study coincided with a previous report [25], and flagella play an important role in infecting host because it increase the motility of bacteria for colonization, or act as adhesive component [26].

The thermolabile hemolysin gene *tlh* was previously used as species specific marker to identify *V. parahaemolyticus* [27]. However in this study, *tlh* recovered from non-*parahaemolyticus* strains showed highly similar sequence with those from *V. parahaemolyticus*. The results indicate that *tlh* cannot be used as species specific marker for detection of *V. parahaemolyticus* due to possible detection of false-positive results. Furthermore, previous study showed an increase in the environmental *V. parahaemolyticus* strains carrying the *tdh* and/or *trh* genes in Malaysia [28]. Interestingly, all isolates in this study lacked the virulence genes associated pathogenic *Vibrio* of human*,* which are the *tdh*, *trh*, *hlyA* and *toxR*_*vc*_ genes, indicating low potential risk for human health. On the other hand, higher percentage of pathogenic *Vibrio* were positive to *trh* (40%) and *tdh* (12.3%) was detected in aquatic animals in other studies [29, 30].

In general, similar virulence genes were widely distributed in the *Harveyi* clade, indicating that the genes are readily transferred among the Vibrionaceae species. This horizontal transferability of virulence genes might be due to their survival benefits in a variety of environments and host organisms [31].

Antibiotics are commonly used in fish farms either as feed additives, prophylaxis or therapy. Oxytetracycline, TE, quinolones, nitrofurans, potentiated sulfonamides, trimethoprim, sarafloxacin, flumequine and oxolinic acid are among the permissible antibiotics that have been used to combat vibriosis [32]. This study revealed that the *Harveyi* clade were highly resistance to AMP and VA. Similarly, all *Vibrio* isolates collected from Malaysian coastal area were resistant to AMP at the rate between 42 and 82% [33], including 100% of isolates from farmed fish [34]. The prevalence of AMP resistance in *Vibrio* isolates from marine environment is generally high, which probably due to the wide usage of AMP. Moreover, resistance to AMP or other penicillin were also well documented from environmental isolated *Vibrio*, ranging from 56 to 100% in China, Italy and U.S. [19, 35, 36].

FEP is one of the newer fourth generation cephalosporins [33]. While none of the isolates in this study showed resistance towards FEP, 84% of the isolates showed immediate sensitive towards this antibiotic. On the other hand, TE and nitrofurans were effective to inhibit the growth of *Vibrio* isolates in this study, thus can be used to treat *Vibrio* infection in Malaysian farm. However, prolonged and misused of antibiotics possess the danger of developing antibiotic resistant genes, that cause the *Vibrio* to develop resistance [13].

In this study, the prevalence of MAR strains of *Vibrio* was at the alarming rate. The results indicate that the MAR strains of *Vibrio* existed widely in the aquaculture farm in this country. Ransangan et al. [34] and You et al. [33] reported high prevalence of multiple antibiotic *Vibrio* recovered from coastal seawater in Malaysia. While there is limited documentation on the information of the use of antibiotics in Malaysian fish farming, emergence of MAR *Vibrio* strains due to excessive utilization of antibiotics has been reported in other countries [2, 19, 37, 38]. For example, high MAR index (0.4) of *Harveyi* clade strains causing scale drop and muscle necrosis disease in groupers was reported in China [19]. Moreover, 77.3% of *V. parahaemolyticus* isolated from oyster in Korea demonstrated MAR to at least three antibiotics, with highest MAR index of 0.75 in one isolate [18].

In general, high prevalence of isolates that were resistant to multiple antibiotics was observed in Pulau Ketam, one of the extensive mariculture farms in Malaysia. While no antibiotic was recorded being used for treatment at our sampling site in Pulau Ketam, the *Vibrio* with multiple antibiotic resistance can be easily transmitted from nearby farms that used antibiotic via water column. In addition, *Vibrio* spp. may acquire and carry antibiotic resistance genes by horizontal genetic transference from and to neighbouring microorganisms. Out of seven sampling site, only the hatchery in Port Dickson reported on the utilization of antibiotic to treat bacterial infection. The rapid increase in antibiotic resistance rendered the treatment to be more difficult. The use of antibiotics in aquaculture also impacts the frequencies of resistance in human pathogens [15, 37]. Therefore, calls for the reduction of antibiotic use has been done worldwide [38, 39].

Other methods of vibriosis control are urgently needed. In recent year, the disruption of quorum sensing has recently been suggested as a cost-effective and environmental friendly method [12]. Several bacteria, micro-algae, macro-algae and aquatic sponges have been shown to inhibit quorum sensing properties in pathogenic *Vibrio* particularly *V. harveyi* [12, 23]. Given the wide distribution of quorum sensing regulated-gene in different *Vibrio* species found in this study, this method are promising to control the expression of virulence factors by different *Vibrio* species in aquaculture. In addition, immunostimulants, bacteriophage, vaccines and probiotics also have potentials to replace antibiotics in controlling and preventing vibriosis in fish farm [40].

## Conclusion

In conclusion, low prevalence of virulence genes was detected in *Vibrio* spp. within the *Harveyi* clade in this study. However, majority of the isolates exhibited multiple resistance to tested antibiotics, highlighting the urgency for reducing the usage of antibiotic in fish farms. It is necessary to perform extensive studies on the spread of antibiotic resistance genes in *Vibrio* to understand the potential risk to public health. In the meantime, alternative non-antibiotic based methods such as quorum quenching and utilization of lytic bacteriophage for preventing and treating bacterial infections in fish farm are needed.

## Methods

### Bacterial strains

Large collection of *Vibrio* strains previously isolated from either healthy or diseased fish cultured in marine farm or hatchery were used in this study. The farm and hatchery included were intensive or semi-intensive farms culturing and producing finfish fry, including Asian seabass (*Lates calcarifer*), red snapper (*Lutjanus* sp.) and hybrid grouper (*Epinephelus* sp.). Seven sampling sites were selected as representative of important fish farming and fry producing area in Peninsular Malaysia; Pulau Ketam and Banting in Selangor, Port Dickson in Negeri Sembilan, Kuala Gula in Perak, Kukup in Johor, Kota Bharu in Kelantan, and Pulau Langkawi in Kedah (Table 1).

**Table 1:** Number of *Vibrio* isolates, type and background information of farms and hatcheries selected in this study

Location	No. of *Vibrio* isolates					Type of sampling sites	Diseases problem encountered	Treatments history	Species of cultured fish	Stocking density
	VA	VP	VH	VC	Total					
Pulau Ketam, Selangor	21	6	8	5	40	Farm	Bacteria, virus, parasite	Chemical	Red snapper, hybrid grouper, Asian seabass, golden pompano	300 fish/cage
Kuala Gula, Perak	3	3	–	1	7	Farm	Bacteria, parasite	Chemical	Red snapper, hybrid grouper, Asian seabass	300 fish/cage
Port Dickson, Negeri Sembilan	2	2	–	2	6	Hatchery	Bacteria, virus	Chemical, antibiotic	Hybrid grouper	400 fish/tank
Pulau Langkawi, Kedah	1	1	–	–	2	Farm	Bacteria, virus	Chemical	Hybrid grouper	1500 fish/cage
Banting, Selangor	–	2	–	–	2	Hatchery	Bacteria	Chemical	Hybrid grouper	NA
Kota Bharu, Kelantan	–	3	–	1	4	Hatchery	Bacteria	Chemical	Hybrid grouper	NA
Kukup, Johor	–	2	–	–	2	Farm	Parasite	Freshwater	Hybrid grouper	1000 fish/cage

*VA Vibrio alginolyticus*, *VP Vibrio parahaemolyticus*, *VH Vibrio harveyi*, *VC Vibrio campbellii*; −: no isolate, *NA* data not available

Identification of the *Vibrio* isolates were verified based on the partial sequencing of *pyrH* as described in previous reports [41, 42]. Based on the recovery rates, only four species of *Vibrio* were selected for this study. A total of 63 isolates representative of *V. alginolyticus*, *V. harveyi*, *V. parahaemolyticus* and *V. campbellii* were analysed. Forty of the isolates were isolated either from Asian seabass, red snapper and hybrid grouper cultured in Pulau Ketam, Selangor. Another 23 *Vibrio* isolates were recovered from hybrid groupers cultured in farm or hatchery located in different states in Peninsular Malaysia (Table 1). The code, species name, source of isolation, clinical sign/s of the host, month and year of isolation, and geographical origin of the isolates as listed in Appendix 1. All isolates were kept in 20% glycerol stock and stored at − 80 °C for further analysis.

### Virulence genes detection

All isolates were sub-cultured from glycerol stock onto Tryptic Soy Agar (TSA) (HiMedia, Mumbai, India), supplemented with 1.5% NaCl and incubated at 30 °C for 18 h. Prior to PCR, genomic DNA of the isolates was extracted using Wizard Genomic DNA Purification Kit (Promega, WI, USA).

A total of nine virulence-associated genes (*chiA*, *vhpA, luxR*, *flaC*, *hlyA*, *toxR*_*vc*_, *tlh*, *tdh* and *trh*) of *Vibrio* were detected by PCR amplification. The sequence of primers used are as listed in Table 2. PCR amplifications were performed in a final volume of 30 μL, which contained 1× PCR buffer, 2 mM MgCl2, 200 uM dNTPs, 10 pmol of forward primer, 10 pmol of reverse primer, 5 U/μL Taq polymerase and 100 ng of template DNA (Promega). The amplification was performed under the following conditions: initial denaturation at 95 °C for 5 min, followed by 30 cycles of 95 °C for 1 min; 50 °C for 1 min (*chiA*, *vhpA* and *luxR*), 55 for 1 min (*flaC*), 60 for 1 min (*hlyA* and *toxR*_*vc*_) and 72 °C for 1 min, and a final extension of 72 °C for 10 min using Eppendorf Mastercycler Nexus Thermal Cycler (Eppendorf, Hamburg, Germany). The amplification of *tlh*, *trh* and *tdh* was performed under the following conditions: initial denaturation at 94 °C for 3 min, followed by 30 cycles of 94 °C for 1 min; 58 °C for 1 min and 72 °C for 1 min, and a final extension of 72 °C for 10 min.

**Table 2:** Virulence factors, sequence of primers, references and expected amplicon size of target gene used in this study

Gene	Virulence factor	Primer sequence (5′-3′)	Reference	Amplicon size (bp)
*chiA*	Chitinase	F: GGAAGATGGCGTGATTGACT R: GGCATCAATTTCCCAAGAGA	[21]	232
*vhpA*	Metalloprotease	F: CTGAACGACGCCCATTATTT R:CGCTGACACATCAAGGCTAA	[21]	201
*luxR*	Quorum sensing factors	F: ATGGACTCAATTGCAAAGAG R: TTAGTGATGTTCACGGTTGT	[21]	618
*flaC*	Flagella of *V. anguillarum*	F: AAATCATTCCAAATCGGTGC R: TCTTTGATTCGGCTCTTA	[25]	580
*hlyA*	Haemolysin of *V. cholera*	F: GGCAAACAGCGAAACAAATAC C R: CTCAGCGGGCTAATACGGTTTA	[48]	738
*toxR*_*Vc*_	Toxin of *V. cholera*	F: ATG TTC GGA TTA GGA CAC R: TAC TCA CAC ACT TTG ATG GC	[49]	883
*tlh*	Thermolabile haemolysin of *V. parahaemolyticus*	F: AAAGCGGATTATGCAGAAGCACTG R: GCTACTTTCTAGCATTTTCTCTGC	[27]	450
*tdh*	Thermostable direct haemolysin (TDH) of *V. parahaemolyticus*	F: GTAAAGGTCTCTGACTTTTGGAC R: TGGAATAGAACCTTCATCTTCACC	[27]	269
*trh*	TDH-related haemolysin (TRH) of *V. parahaemolyticus*	F: TTGGCTTCGATATTTTCAGTATCT R: CATAACAAACATATGCCCATTTCCG	[27]	500

Amplified PCR products were visualised on 1.2% agarose gel stained with ethidium bromide, run at 90 V for 40 min, and photographed using a gel documented system. The confirmation of the presence of genes were by partial sequencing (FirstBase, Kuala Lumpur, Malaysia) and BLAST comparison with GenBank (http://blast.ncbi.nlm.nih.gov/). Following multiple alignment of genes with their closed taxa by CLUSTAL W method, neighbour-joining trees were constructed using the Kimura 2-parameter model with MEGA version 7.0 with bootstraps of 1000 replicates [43].

### Antibiotic sensitivity testings

The antibiotics sensitivity of the isolates were examined by the disc diffusion methods [44]. A total of 18 representative antimicrobial agents (Oxoid, London, UK), including penicillins (ampicillin (AMP): 10 μg; penicillin G (P): 10 units), cephems (cefotaxime (CTX): 30 μg; cefepime (FEP): 30 μg; cephalothin (KF): 30 μg), aminoglycosides (gentamycin (CN): 10 μg; kanamycin (K): 30 μg; streptomycin (S): 10 μg), and others such as nalidixic acid (NA): 30 μg; trimethoprim/sulfamethoxazole (SXT): 1.25/23.75 μg; chloramphenicol (C): 30 μg; nitrofurantoin (F): 300 μg; ciprofloxacin (CIP): 5 μg; tetracycline (TE): 30 μg; erythromycin (E): 15 μg; and vancomycin (VA): 30 μg were used.

Following incubation for 18–24 h, the isolates were then inoculated in sterile saline water to achieve turbidity equivalent to 0.5 MacFarland standard. The broth were evenly swabbed onto Mueller Hinton agar (HiMedia) supplemented with 1% of NaCl [45]. Antibiotic discs were aseptically placed on the swabbed plates. The plates were then incubated at 35 °C for 16–18 h, and the clearing zone was recorded. Testing was confirmed in duplicate. The resistance profiles (resistant, intermediate or susceptible) were assigned using criteria described by CLSI [44, 46] and Bauer et al. [47]. The multiple antibiotic resistance (MAR) index was determined for each isolate [20]. Table 3 summarized the list of antibiotics and the zone diameter interpretive criteria used in this study.

**Table 3:** List of antibiotics used in this study

Antimicrobial class	Antimicrobial agent	Code	Dose	Zone diameter interpretive criteria		
				Sensitive	Intermediate	Resistance
*Cell envelope antibiotics*						
Penicillins	Ampicillin	AMP	10 μg	≥17	14–16	13
	Penicillin G	P	10 unit	≥29	21–28	≤20
Cephems	Cefotaxime	CTX	30 μg	≥26	19–24	≤18
	Cefepime	FEP	30 μg	≥25	19–24	≤18
	Cephalothin	KF	30 μg	≥18	15–17	≤14
*Protein synthesis inhibitors*						
Aminoglycosides	Gentamycin	CN	10 μg	≥15	12–14	≤11
	Kanamycin	K	30 μg	≥18	14–17	≤13
	Streptomycin	S	10 μg	≥15	12–14	≤11
Tetracyclines	Tetracycline	TE	30 μg	≥		≤
*Nucleic acid inhibitors*						
Quinolones	Ciprofloxacin	CIP	5 μg	≥21	16–20	≤15
	Nalidixic acid	NA	30 μg	≥19	14–18	≤13
DNA inhibitors	Nitrofurantoin	F	300 μg	≥17	15–16	≤14
Potentiated sulfonamides	Sulfamethoxazole/trimethoprim	SXT	1.25/23.75 μg	≥16	11–15	≤10
*Phenicol derivatives*						
Chloramphenicols	Chloramphenicol	C	30 μg	≥18	13–17	≤12
*Transpeptidation/Translocation*						
Macrolides	Erythromycin	E	15 μg	≥18	14–17	≤13
*Glycopeptide*						
Polypeptides	Vancomycin	VA	30 μg	≥12	10–11	≤9

Zone diameter interpretive criteria were referred to the performance standards proposed by Clinical and Laboratory Standards Institute [44, 46], with the exception for erythromycin and vancomycin which referred to Bauer et al. [47]

## Appendix

**Table 4:** Details on list of isolates used in this study

No.	Code	Species	Source of isolation	Clinical sign/s of host	Month and year of isolation	Geographical origin
1	SNA 92-L	*V. A*	Liver of red snapper	Rotten caudal fin, blind left eye and pale liver	Dec 2016	Pulau Ketam, Selangor
2	GRO 99-E	*V. A*	Eye of hybrid grouper	Lesion on body, haemorrhagic liver and kidney and rotten caudal fin	Dec 2016	Pulau Ketam, Selangor
3	GRO 102-K	*V. A*	Kidney of hybrid grouper	Blind left eye and pale liver	Jan 2017	Pulau Ketam, Selangor
4	SEA 107-S	*V. A*	Skin mucus of Asian seabass	Haemorrhagic liver	Jan 2017	Pulau Ketam, Selangor
5	SNA 109-S1	*V. A*	Skin mucus of red snapper	Haemorrhagic liver and kidney	Jan 2017	Pulau Ketam, Selangor
6	SEA 144-L	*V. A*	Liver of Asian seabass	Pale liver and haemorrhagic kidney	Jan 2017	Pulau Ketam, Selangor
7	SNA 144-L	*V. A*	Liver of red snapper	Blind left eye, pale liver and pale kidney	Feb 2017	Pulau Ketam, Selangor
8	GRO 144-E1	*V. A*	Eye of hybrid grouper	Lesion on body, blind left eye and haemorrhagic kidney	Feb 2017	Pulau Ketam, Selangor
9	GRO 162-S	*V. A*	Skin mucus of hybrid grouper	Pale liver	Feb 2017	Pulau Ketam, Selangor
10	SNA 165-E	*V. A*	Eye of red snapper	Pale liver	Jan 2017	Pulau Ketam, Selangor
11	GRO 198-S1	*V. A*	Skin lesion on hybrid grouper	Lesion on pectoral fin	Apr 2017	Pulau Ketam, Selangor
12	SNA 199-E1	*V. A*	Eye of red snapper	No symptom	Apr 2017	Pulau Ketam, Selangor
13	SEA 205-E1	*V. A*	Eye of Asian seabass	No symptom	May 2017	Pulau Ketam, Selangor
14	GRO 208-K1	*V. A*	Kidney of hybrid grouper	Lesion on all fins	May 2017	Pulau Ketam, Selangor
15	SNA 212-S1	*V. A*	Skin mucus of red snapper	Bulging eye	May 2017	Pulau Ketam, Selangor
16	GRO 223-S	*V. A*	Skin mucus of hybrid grouper	Lesion on body and all fins, and haemorrhagic liver	May 2017	Pulau Ketam, Selangor
17	SNA 226-E	*V. A*	Eye of red snapper	Haemorrhage liver	May 2017	Pulau Ketam, Selangor
18	SNA 266-S	*V. A*	Skin mucus on red snapper	Rotten caudal fin and pale liver	May 2017	Pulau Ketam, Selangor
19	GRO 268-E	*V. A*	Eye of hybrid grouper	Haemorrhagic liver	May 2017	Pulau Ketam, Selangor
20	GRO 286-L1	*V. A*	Liver of hybrid grouper	Ulcer on body and haemorrhagic liver	Aug 2017	Pulau Ketam, Selangor
21	SNA 297-S1	*V. A*	Skin mucus of red snapper	No symptom	Aug 2017	Pulau Ketam, Selangor
22	PKG28	*V. A*	Spleen of hybrid grouper	Lesion on operculum and fins	Nov 2017	Kuala Gula, Perak
23	PKGS29	*V. A*	Spleen of hybrid grouper	Lesion on operculum	Nov 2017	Kuala Gula, Perak
24	PKG29	*V. A*	Liver of hybrid grouper	Lesion on operculum	Mar 2017	Kuala Gula, Perak
25	NL24	*V. A*	Liver of hybrid grouper	Lesion on body and fins, and pale liver	Dis 2017	Port Dickson, Negeri Sembilan
26	NS24	*V. A*	Spleen of hybrid grouper	Lesion on body and fins, and pale liver	Dis 2017	Port Dickson, Negeri Sembilan
27	LL6	*V. A*	Liver of hybrid grouper	No symptom	Mar 2017	Pulau Langkawi, Kedah
28	SNA 147-L2	*V.P*	Liver of red snapper	Haemorrhagic liver and kidney	Feb 2017	Pulau Ketam, Selangor
29	GRO 180-K1	*V.P*	Kidney of hybrid grouper	No symptom	Apr 2017	Pulau Ketam, Selangor
30	SEA 196-S1	*V.P*	Skin lesion on Asian seabass	Lesion on body	Apr 2017	Pulau Ketam, Selangor
31	GRO 251-K1	*V.P*	Kidney of hybrid grouper	Ulcer on body and fins, and pale liver	July 2017	Pulau Ketam, Selangor
32	SEA 252-E1	*V.P*	Eye of Asian seabass	Rotten caudal fin	July 2017	Pulau Ketam, Selangor
33	GRO 286-E1	*V.P*	Eye of hybrid grouper	Ulcer on body and haemorrhagic liver	Aug 2017	Pulau Ketam, Selangor
34	BK2G2	*V.P*	Kidney of hybrid grouper	Lesion on operculum, pale liver and enlarged spleen	Nov 2016	Kuala Gula, Perak
35	BK3G2	*V.P*	Kidney of hybrid grouper	Pale liver	Nov 2016,	Banting, Selangor
36	PKGL4	*V.P*	Liver of hybrid grouper	Lesion on body	Nov 2017	Kuala Gula, Perak
37	PKGS11	*V.P*	Spleen of hybrid grouper	No symptom	Nov 2017	Kuala Gula, Perak
38	PKGL27	*V.P*	Liver of hybrid grouper	Lesion on fins	Nov 2017	Kuala Gula, Perak
39	NK6	*V.P*	Kidney of hybrid grouper	Lesion on body and fins, and pale liver	Dis 2017	Port Dickson, Negeri Sembilan
40	NS8	*V.P*	Spleen of hybrid grouper	Lesion on body and fins	Dis 2017	Port Dickson, Negeri Sembilan
41	LK14	*V.P*	Kidney of hybrid grouper	No symptom	Aug 2017	Pulau Langkawi, Kedah
42	KK3	*V.P*	Kidney of hybrid grouper	Lesion on operculum, and enlarged spleen and liver	Sept 2017	Kota Bharu, Kelantan
43	KL4	*V.P*	Liver of hybrid grouper	Lesion on body, and enlarged spleen and liver	Sept 2017	Kota Bharu, Kelantan
44	KL17	*V.P*	Liver of hybrid grouper	Lesion on body, eye opacity, pale liver and enlarged spleen	Sept 2017	Kota Bharu, Kelantan
45	JS14	*V.P*	Spleen of hybrid grouper	No symptom	Dis 2017	Kukup, Johor
46	JS24	*V.P*	Spleen of hybrid grouper	No symptom	Dis 2017	Kukup, Johor
47	SEA 131-K1	*V.H*	Kidney of Asian seabass	Haemorrhagic kidney and liver	Feb 2017	Pulau Ketam, Selangor
48	SNA 143-L1	*V.H*	Liver of red snapper	Rotten caudal fin and haemorrhagic kidney	Feb 2017	Pulau Ketam, Selangor
49	GRO 154-E1	*V.H*	Eye of hybrid grouper	Lesion on pelvic fin and severely pale liver	Mar 2017	Pulau Ketam, Selangor
50	SEA 166-E	*V.H*	Eye of Asian seabass	Lesion on body and haemorrhagic liver	Apr 2017	Pulau Ketam, Selangor
51	SEA 192-S1	*V.H*	Skin mucus of Asian seabass	Haemorrhagic liver	Apr 2017	Pulau Ketam, Selangor
52	GRO 200-L2	*V.H*	Liver of hybrid grouper	No symptom	Apr 2017	Pulau Ketam, Selangor
53	SEA 207-S1	*V.H*	Skin mucus of Asian seabass	Haemorrhagic liver and kidney	May 2017	Pulau Ketam, Selangor
54	SNA 272-S2	*V.H*	Skin mucus of red snapper	Pale liver	July 2017	Pulau Ketam, Selangor
55	SEA 178-K1	*V. C*	Kidney of Asian seabass	Haemorrhagic liver and kidney	Apr 2017	Pulau Ketam, Selangor
56	GRO 186-K1	*V. C*	Kidney of hybrid grouper	Lesion of all fins	Apr 2017	Pulau Ketam, Selangor
57	SNA 244-S2	*V. C*	Skin mucus of red snapper	Haemorrhagic liver and kidney	June 2017	Pulau Ketam, Selangor
58	SEA 206-S1	*V. C*	Skin mucus of Asian seabass	Haemorrhage liver	May 2017	Pulau Ketam, Selangor
59	GRO 230-L1	*V. C*	Liver of hybrid grouper	Lesion on whole body, pale liver and haemorrhage kidney	June 2017	Pulau Ketam, Selangor
60	PKGL28	*V. C*	Liver of hybrid grouper	Lesion on operculum and dorsal fin	Nov 2017	Kuala Gula, Perak
61	NS26	*V. C*	Spleen of hybrid grouper	Lesion on body and discoloration	Dis 2017	Port Dickson, Negeri Sembilan
62	NS12	*V. C*	Spleen of hybrid grouper	Lesion on body and fins	Dis 2017	Port Dickson, Negeri Sembilan
63	KL28	*V. C*	Liver of hybrid grouper	Lesion on body and fins, pale liver and spleen enlarged	Sept 2017	Kota Bharu, Kelantan

**V.A*: *Vibrio alginolyticus*; *V.P*: *Vibrio parahaemolyticus*; *V.P*: *Vibrio harveyi*; *V.C*: *Vibrio campbelli*

## Abbreviations

AMP: Ampicillin
C: Chloramphenicol
CIP: Ciprofloxacin
CN: Gentamycin
CTX: Cefotaxime
E: Erythromycin
F: Nitrofurantoin
FEP: Cefepime
K: Kanamycin
KF: Cephalothin
MAR: Multiple antibiotic resistance
NA: Nalidixic acid
P: Penicillin G
S: Streptomycin
SXT: Trimethoprim/sulfamethoxazole
TE: Tetracycline
TSA: Tryptic soy agar
VA: Vancomycin
